# Predicting 6-month decisional regret among family surrogate decision-makers of ICU patients: development and internal validation of prediction models

**DOI:** 10.3389/fpubh.2026.1928359

**Published:** 2026-09-03

**Authors:** Xiaoyan Gong, Weijing Sui, Chuchu Zhang, Chaoyi Zhang, Yiyu Zhuang

**Affiliations:** Nursing Department, Sir Run Run Shaw Hospital, Zhejiang University School of Medicine, Hangzhou, Zhejiang, China

**Keywords:** decisional regret, surrogate decision-makers, intensive care units, prediction model, machine learning, trust in physicians, health literacy

## Abstract

**Aim:**

To develop and internally validate prediction models estimating the individual probability of high decisional regret at 6 months among family surrogate decision-makers (FSDMs) of intensive care unit (ICU) patients, and to compare regression-based and machine-learning approaches with respect to discrimination, calibration and clinical utility.

**Design:**

A single-center prospective cohort study for the development and internal validation of prediction models.

**Methods:**

This single-center observational prediction-model study was conducted in five ICUs of a tertiary university-affiliated hospital in Hangzhou, China, from October 2023 to October 2024. We enrolled 336 family surrogate decision-makers. Sociodemographic characteristics, health literacy, eHealth literacy, anxiety, depression, trust in physicians, and family functioning were collected within 3 days after ICU discharge. Decisional regret was assessed 6 months after hospital discharge using the Decision. Regret Scale and analyzed as a binary outcome, with a transformed score of ≥25 defining high decisional regret. Candidate predictors were selected using univariate analysis and least absolute shrinkage and selection operator regression. Logistic regression, random forest, support vector machine, and extreme gradient boosting models were developed and internally validated using a 70:30 train-test split. Model performance was assessed by discrimination, calibration, and decision curve analysis. SHAP analysis was used as an exploratory interpretability method.

**Results:**

Among 336 family surrogate decision-makers, 165 (49.11%) had high decisional regret and 171 (50.89%) had low decisional regret. Univariate analysis identified nine variables associated with decisional regret, including health literacy, trust in physicians, patient survival status, relationship to the patient, household income, anxiety, depression, and family functioning (all *p* < 0.05). In the test set, logistic regression showed the strongest overall performance (AUC 0.875, 95% CI 0.808–0.942; accuracy 0.752; sensitivity 0.702; specificity 0.796; F1 score 0.725). It also showed the most stable calibration and clinical net benefit. The final model comprised five predictors: eHealth literacy, trust in physicians, average monthly household income, anxiety and depression. In a secondary explanatory analysis, each contributed independently to the predicted probability (all *p* < 0.05); these estimates are reported to aid interpretation of the model and are not intended as causal effect estimates. Exploratory SHAP analysis identified trust in physicians, anxiety, and eHealth literacy as prominent contributors to model predictions.

**Conclusion:**

Decisional regret was common among ICU family surrogate decision-makers. Trust in physicians, eHealth literacy, household income, anxiety, and depression were key predictors. Among the candidate models, logistic regression provided the best balance of discrimination, calibration, and clinical utility, suggesting that a parsimonious and interpretable model may support early risk screening in ICU family-centered care.

## Introduction

Decision-making capacity, defined as the ability to understand relevant information, appreciate illness and its consequences, reason about treatment options, and communicate a choice, is often impaired during critical illness. Because many ICU patients cannot make treatment decisions for themselves, decisions are commonly guided by advance directives or made by family surrogate decision-makers (1, 2). In China, the uptake of advance directives remains low (3, 4), and ICU decisions are often led by family members. This context places relatives at the center of high-stakes treatment decisions.

ICU decisions are frequent, time-sensitive, and emotionally demanding. Family surrogates may need to decide about emergency surgery, escalation of life-sustaining treatments, or limitation and withdrawal of treatment before they fully understand the patient’s condition and prognosis. These experiences have been associated with anxiety, depression, and post-traumatic stress symptoms among family members (5, 6). Decisional regret is a common cognitive and emotional response after difficult health decisions. It involves distress, guilt, remorse, and counterfactual thinking about whether another option might have produced a better outcome (7, 8). Because decisional regret reflects both the decision-making process and the perceived outcome, it is widely used to evaluate the quality and longer-term consequences of health-related decisions (9–12).

Our prior review (13) showed that ICU family surrogate decision-makers experience substantial emotional and cognitive burdens. The socio-ecological model provides a useful framework for understanding these burdens because it considers factors at multiple levels. At the individual level, medical knowledge, health literacy, anxiety, and depression may shape how surrogates understand information and appraise risk. At the family level, family functioning and support may influence coping resources. At the healthcare system level, trust between family members and physicians may affect information exchange and shared decision-making. These factors are likely to interact in shaping decisional regret.

Quantitative studies have linked decisional regret with discrepancies between expected and actual decision-making roles (14), time pressure and informational asymmetry (15), insufficient support (16), and perceived deterioration in long-term quality of life (17). Other studies have reported associations between decisional regret and depression, anxiety, sex, and postoperative complications in surgical or treatment-decision settings (18–21). However, most evidence has focused on single or relatively distal factors (22). Few studies have used a theory-driven, cross-level framework to quantify modifiable determinants among ICU family surrogate decision-makers. In addition, prediction models tailored to this population remain scarce, and regression-based and machine-learning approaches have rarely been compared for this outcome.

It should be noted that, risk-factor analysis and prediction modeling answer different questions. Risk-factor analysis estimates the average association between an exposure and an outcome across a population, conditional on covariates; it does not indicate the probability of the outcome for the individual in front of the clinician. Prediction modeling combines multiple predictors into a single estimated probability for an individual and is judged by discrimination, calibration and clinical usefulness rather than by the magnitude or statistical significance of individual coefficients. This distinction is consequential in the ICU. Decisional regret cannot be measured at the moment the decision is made, and by the time it is detectable, at 6 months, it is frequently entrenched and resistant to intervention. What clinicians require at ICU discharge is therefore not a population-level list of correlates but a prospective means of identifying which surrogates are likely to develop regret, so that enhanced communication, structured decision support and timely psychological referral can be offered while these measures may still alter the trajectory. All predictors retained in our final model can be assessed with brief validated instruments during the ICU stay, which makes such screening feasible in routine practice. To our knowledge, no prediction model for decisional regret among ICU family surrogate decision-makers has been reported, and regression-based and machine-learning approaches have not previously been compared for this outcome.

In conclusion, guided by a socio-ecological framework, this study aimed to develop and internally validate prediction models for high decisional regret at 6 months among ICU family surrogate decision-makers, and to compare regression-based and machine-learning approaches with respect to discrimination, calibration, clinical utility and interpretability. The framework was used to ensure that candidate variables spanned the individual, interpersonal/family, healthcare-system and socioeconomic levels; final predictors were selected empirically.

## Methods

### Study design and setting

This study was conducted in accordance with the Declaration of Helsinki and relevant national and institutional guidelines. The study protocol was reviewed and approved by the institutional ethics committee (Approval No. 2024-Research-0429). Written informed consent was obtained from all participants.

This single-center prospective cohort study, conducted for the purpose of developing and internally validating prediction models, was carried out from October 2023 to October 2024. Participants were recruited from five ICUs of a 4,300-bed tertiary university-affiliated hospital in Zhejiang, China. Predictors were measured within 3 days after ICU discharge, which corresponds to the intended moment of model use in practice. The outcome was measured 6 months after hospital discharge. No outcome information was available at the time of predictor measurement, and outcome assessors were blinded to predictor values. The prediction-model component was guided by TRIPOD+AI reporting recommendations (23), and the observational reporting followed the Strengthening the Reporting of Observational Studies in Epidemiology (STROBE) guidelines (24).

### Study population

Family surrogate decision-makers (FSDMs) of ICU patients were recruited according to predefined eligibility criteria. Individuals were eligible if they were at least 18 years old, were the patient’s legally authorized medical decision-maker, and were a legal relative of the patient, such as a spouse, parent, adult child, or sibling. To ensure a close emotional relationship with the patient, participants were also required to score at least 8 on the emotional dependence and overall dependence subscales of the Partner Dependence Scale (25). Participants were enrolled only if they were willing to participate and able to provide written informed consent. We excluded individuals who could not communicate in Chinese or who belonged to a vulnerable group, including people with diagnosed mental disorders, cognitive impairment, critical illness themselves, minors, pregnant women, and those who were illiterate. Participants could withdraw at any time, and investigators could discontinue participation if continued involvement was considered inappropriate or not in the participant’s best interests.

### Measurements

1. General information questionnaire

Sociodemographic characteristics were collected using a structured questionnaire and included sex, age, religion, educational attainment, marital status, and employment status or occupation.

(1) Health literacy measures

The Functional, Communicative and Critical Health Literacy Scale (FCCHL) assesses three dimensions of health literacy: functional, communicative, and critical health literacy (26, 27). Functional health literacy reflects basic reading and writing skills. Communicative health literacy reflects the ability to obtain and discuss health information. Critical health literacy reflects the ability to evaluate information and use it for health decisions. The scale comprises 14 items rated on a 4-point Likert scale, with items in the functional health literacy subscale reverse scored. Higher scores indicate higher health literacy. The Chinese version has shown good reliability and validity, including a mean scale-level content validity index of 0.965, a test–retest reliability coefficient of 0.715, and a Cronbach’s alpha of 0.891 for the total scale (27).

The eHealth Literacy Scale (eHEALS) assesses perceived ability to search for, locate, understand, appraise, and apply health information from electronic resources (28). The Chinese version contains eight items rated on a 5-point Likert scale from 1 (strongly disagree) to 5 (strongly agree), yielding a total score from 8 to 40. Higher scores indicate higher eHealth literacy. The Chinese version has demonstrated good reliability and validity, with a Cronbach’s alpha of 0.96, McDonald’s omega of 0.92, and split-half reliability of 0.96 (29).

(2) Hospital Anxiety and Depression Scale (HADS)

The Hospital Anxiety and Depression Scale (HADS) is a 14-item self-report measure of anxiety and depressive symptoms (30). It includes two seven-item subscales: HADS-A for anxiety and HADS-D for depression. Each item is rated from 0 to 3, and each subscale ranges from 0 to 21. Scores of 0–7 are considered normal, 8–10 indicate possible caseness, and 11–21 indicate definite caseness. Higher scores indicate more severe symptoms. The Chinese version has been used in hospital populations and has shown acceptable reliability and validity (31).

(3) Wake Forest Physician Trust Scale

The Wake Forest Physician Trust Scale measures trust in physicians across dimensions of benevolence and technical competence (32). It includes 10 items related to physicians’ caring attitude, communication, clinical skills, and expertise. Higher scores indicate greater trust in physicians. Previous studies have reported good reliability for the scale.

(5) Family APGAR Index

The Family APGAR Index assesses perceived family support across five dimensions: adaptation, partnership, growth, affection, and resolve (33, 34). Each item is rated on a 3-point scale from 0 to 2, yielding a total score from 0 to 10. Scores of 0–3 indicate severe family dysfunction, 4–6 indicate moderate dysfunction, and 7–10 indicate good family functioning. Higher scores represent better family functioning. The Chinese version has been widely used among patients with chronic diseases and their families and has shown good reliability and validity (35, 36).

(6) Decision Regret Scale (DRS)

The Decision Regret Scale (DRS) is a five-item instrument developed to assess regret after health-related decisions (8). Items are rated on a 5-point Likert scale, with items 2 and 4 reverse scored. Total scores are transformed to a 0–100 scale, with higher scores indicating greater regret. Previous studies have commonly used a score above 25 to indicate moderate-to-severe or relatively high decisional regret (37, 38). In this study, a DRS score of at least 25 was defined as high decisional regret, and a score below 25 was defined as low decisional regret. The Chinese version has shown good psychometric properties, with Cronbach’s alpha coefficients ranging from 0.81 to 0.83 (37).

### Outcome definition

The outcome was decisional regret at 6 months after hospital discharge, measured with the Decision Regret Scale (DRS) and analyzed as a binary variable. In accordance with previous studies (37, 38), a transformed DRS score of ≥25 was defined as high decisional regret and a score of <25 as low decisional regret. A binary outcome was chosen for two reasons. First, the intended clinical use of the model is dichotomous: to identify FSDMs who should be offered additional communication and psychological support. Second, the threshold of 25 has been applied consistently in the decisional-regret literature, which supports comparability across studies.

### Data collection

Before formal data collection, a pilot survey was conducted to assess questionnaire completion time, readability, and item clarity. Thirty family members of critically ill patients were recruited by convenience sampling according to the same eligibility criteria, and written informed consent was obtained. The questionnaire was administered through the Wenjuanxing online survey platform. Family members completed the survey by scanning a QR code in the ICU nursing units. The research team assessed whether any items were ambiguous or difficult to understand and refined the questionnaire based on pilot feedback.

During formal data collection, trained investigators contacted FSDMs within 3 days after the patient was transferred out of the ICU, either by telephone or face to face. After informed consent was obtained, participants completed a structured questionnaire covering patient and family sociodemographic characteristics, health literacy, eHealth literacy, anxiety, depression, family functioning, and trust in physicians. Patient clinical outcomes were monitored through the hospital electronic information system, and the date of hospital discharge was recorded. Six months after discharge, FSDMs were contacted by telephone to assess decisional regret related to treatment decisions. What calls for special attention is that, for FSDMs whose patient had died during the follow-up period, the 6-month assessment was conducted by investigators trained in bereavement communication. These participants were reminded that they could decline any question, pause, or withdraw at any point, and the interview was rescheduled or terminated if signs of acute distress were observed.

### Statistical analysis

All analyses were performed in R (version 4.5.1). Continuous variables were summarized as median and interquartile range (IQR), and categorical variables were summarized as frequency and percentage. The rank-sum test was used for non-normally distributed continuous variables, and the chi-square test was used for categorical variables.

Four candidate prediction approaches were compared: logistic regression (LR), random forest (RF), support vector machine (SVM), and extreme gradient boosting (XGBoost). The main R packages used included pROC, ggplot2, rms, survival, tidyverse, xgboost, and vip.

### Model development and validation

#### Feature selection and model building

To improve predictive performance and reduce overfitting, candidate predictors were screened using univariate analyses and least absolute shrinkage and selection operator (LASSO) regression. Candidate predictors were screened in two steps. First, univariate analyses were used to describe differences between the high and low decisional regret groups; these analyses were descriptive and were not used to include or exclude variables. Second, LASSO regression with 10-fold cross-validation was applied to all candidate variables, and the variables with non-zero coefficients at *λ*.min were retained as the final predictor set. The same predictor set was used for all four modeling approaches (LR, RF, SVM and XGBoost), so that differences in performance reflect differences in algorithm rather than in input features. The dataset was randomly split into a training set (70%) and a test set (30%). Candidate prediction models were then developed using LR, RF, SVM, and XGBoost. Four modeling approaches were compared because no prediction model for decisional regret in this population had previously been reported, and it was not known *a priori* whether the associations between candidate predictors and the outcome were linear and additive. Random forest and XGBoost can capture non-linear effects and interactions without prior specification, and support vector machines can accommodate non-linear decision boundaries; comparing these with logistic regression therefore provided a test of whether additional model complexity was warranted. Logistic regression was pre-specified as the reference model on the grounds of interpretability and ease of clinical implementation, and the more complex algorithms were regarded as justified only if they improved out-of-sample performance.

#### Model performance

Model performance was assessed in both the training and test sets. Discrimination was evaluated using the area under the receiver operating characteristic curve (AUC), accuracy, sensitivity, specificity, and F1 score. Calibration was assessed using calibration curves. Clinical utility was evaluated using decision curve analysis (DCA).

#### Model interpretability

SHapley Additive exPlanations (SHAP) analysis was conducted as an exploratory interpretability analysis of the XGBoost model to examine the relative contribution and direction of candidate predictors.

## Results

### Baseline characteristics

A total of 400 questionnaires were collected from ICU family members. After 64 questionnaires with more than 30% missing data were excluded, 336 participants were included in the final analysis. At 6 months after hospital discharge, 165 participants (49.11%) were classified into the high decisional regret group and 171 (50.89%) into the low decisional regret group.

### Univariate analysis of decisional regret

Nine variables differed significantly between the high and low decisional regret groups (all *p* < 0.05; Tables 1, 2): FCCHL score, eHealth literacy, trust in physicians, patient 6-month survival status, relationship to the patient, average monthly household income, anxiety, depression and family functioning. The high decisional regret group had lower health literacy and trust in physicians, a higher proportion of patient death, and higher proportions of low income, anxiety, depression, and family dysfunction. Age, sex, educational level, occupation, marital status, and co-residence with the patient did not differ significantly between groups (all *p* > 0.05).

**Table 1:** Baseline characteristics of ICU family surrogate decision-makers and patients according to decisional regret status.

Variable	Total (*N* = 336)^1^	Low regret (*n* = 171)^1^	High regret (*n* = 165)^1^	*p* value^2^
Age, years				>0.9
Median (Q1, Q3)	46.0 (38.0, 54.0)	46.0 (37.0, 55.0)	46.0 (39.0, 54.0)	
FCCHL score				<0.001
Median (Q1, Q3)	46.0 (43.0, 48.0)	47.0 (45.0, 49.0)	45.0 (43.0, 48.0)	
eHealth literacy score				<0.001
Median (Q1, Q3)	35.0 (33.0, 36.0)	35.0 (34.0, 36.0)	34.0 (33.0, 35.0)	
Trust in physicians score				<0.001
Median (Q1, Q3)	68.0 (48.0, 80.0)	78.0 (66.0, 80.0)	54.0 (42.0, 70.0)	
Survival status of patients within 6 months after hospital discharge				<0.001
Alive	239.0 (71.1%)	150.0 (87.7%)	89.0 (53.9%)	
Deceased	97.0 (28.9%)	21.0 (12.3%)	76.0 (46.1%)	
Primary diagnosis				0.057
Injury/Poisoning	23.0 (6.8%)	14.0 (8.2%)	9.0 (5.5%)	
Digestive	85.0 (25.3%)	36.0 (21.1%)	49.0 (29.7%)	
Respiratory	41.0 (12.2%)	15.0 (8.8%)	26.0 (15.8%)	
Nervous	82.0 (24.4%)	48.0 (28.1%)	34.0 (20.6%)	
Circulatory	71.0 (21.1%)	42.0 (24.6%)	29.0 (17.6%)	
Genitourinary	11.0 (3.3%)	3.0 (1.8%)	8.0 (4.8%)	
Infectious	19.0 (5.7%)	11.0 (6.4%)	8.0 (4.8%)	
Endocrine	4.0 (1.2%)	2.0 (1.2%)	2.0 (1.2%)	
Relationship to patient				0.021
Sibling	15.0 (4.5%)	9.0 (5.3%)	6.0 (3.6%)	
Spouse	109.0 (32.4%)	67.0 (39.2%)	42.0 (25.5%)	
Child/Child-in-law	188.0 (56.0%)	87.0 (50.9%)	101.0 (61.2%)	
Parent	16.0 (4.8%)	4.0 (2.3%)	12.0 (7.3%)	
Grandchild	8.0 (2.4%)	4.0 (2.3%)	4.0 (2.4%)	
Sex				0.8
Male	173.0 (51.5%)	89.0 (52.0%)	84.0 (50.9%)	
Female	163.0 (48.5%)	82.0 (48.0%)	81.0 (49.1%)	
Educational level				0.4
High school/vocational	86.0 (25.6%)	48.0 (28.1%)	38.0 (23.0%)	
Primary/junior high	148.0 (44.0%)	70.0 (40.9%)	78.0 (47.3%)	
College/university+	102.0 (30.4%)	53.0 (31.0%)	49.0 (29.7%)	
Occupation				>0.9
Retired	57.0 (17.0%)	29.0 (17.0%)	28.0 (17.0%)	
Company employee	84.0 (25.0%)	42.0 (24.6%)	42.0 (25.5%)	
Public servant	28.0 (8.3%)	13.0 (7.6%)	15.0 (9.1%)	
Manual worker	20.0 (6.0%)	12.0 (7.0%)	8.0 (4.8%)	
Farmer	15.0 (4.5%)	8.0 (4.7%)	7.0 (4.2%)	
Self-employed	72.0 (21.4%)	36.0 (21.1%)	36.0 (21.8%)	
Healthcare/education	11.0 (3.3%)	6.0 (3.5%)	5.0 (3.0%)	
Student/unemployed	49.0 (14.6%)	25.0 (14.6%)	24.0 (14.5%)	
Marital status				0.5
Single/divorced/widowed	41.0 (12.2%)	23.0 (13.5%)	18.0 (10.9%)	
Married	295.0 (87.8%)	148.0 (86.5%)	147.0 (89.1%)	
Average monthly household income				0.003
≥10,000 CNY	72.0 (21.4%)	48.0 (28.1%)	24.0 (14.5%)	
<10,000 CNY	264.0 (78.6%)	123.0 (71.9%)	141.0 (85.5%)	
Co-residence with patient				0.2
No	176.0 (52.4%)	83.0 (48.5%)	93.0 (56.4%)	
Yes	160.0 (47.6%)	88.0 (51.5%)	72.0 (43.6%)	
Perceived burden				0.066
None/slight	216.0 (64.3%)	118.0 (69.0%)	98.0 (59.4%)	
Moderate/heavy	120.0 (35.7%)	53.0 (31.0%)	67.0 (40.6%)	
Anxiety present				<0.001
No	239.0 (71.1%)	141.0 (82.5%)	98.0 (59.4%)	
Yes	97.0 (28.9%)	30.0 (17.5%)	67.0 (40.6%)	
Depression present				<0.001
No	138.0 (41.1%)	96.0 (56.1%)	42.0 (25.5%)	
Yes	198.0 (58.9%)	75.0 (43.9%)	123.0 (74.5%)	
Family dysfunction				<0.001
Good	283.0 (84.2%)	159.0 (93.0%)	124.0 (75.2%)	
Dysfunctional	53.0 (15.8%)	12.0 (7.0%)	41.0 (24.8%)	

1 Median (IQR) for continuous variables; *n* (%) for categorical variables.

2 Wilcoxon rank sum test; Pearson’s Chi-squared test; NA; Fisher’s exact test.

**Table 2:** Mapping table.

Socio-ecological level	Construct	Instrument
Individual	Health literacy	FCCHL
Individual	eHealth literacy	eHEALS
Individual	Anxiety	HADS-A
Individual	Depression	HADS-D
Interpersonal/Family	Family functioning	Family APGAR Index
Interpersonal/Family	Relationship to the patient	General information questionnaire
Healthcare system	Trust in physicians	Wake Forest Physician Trust Scale
Socioeconomic status	Average monthly household income、education、occupation	General information questionnaire

### LASSO-based predictor selection

**Figure 1:**
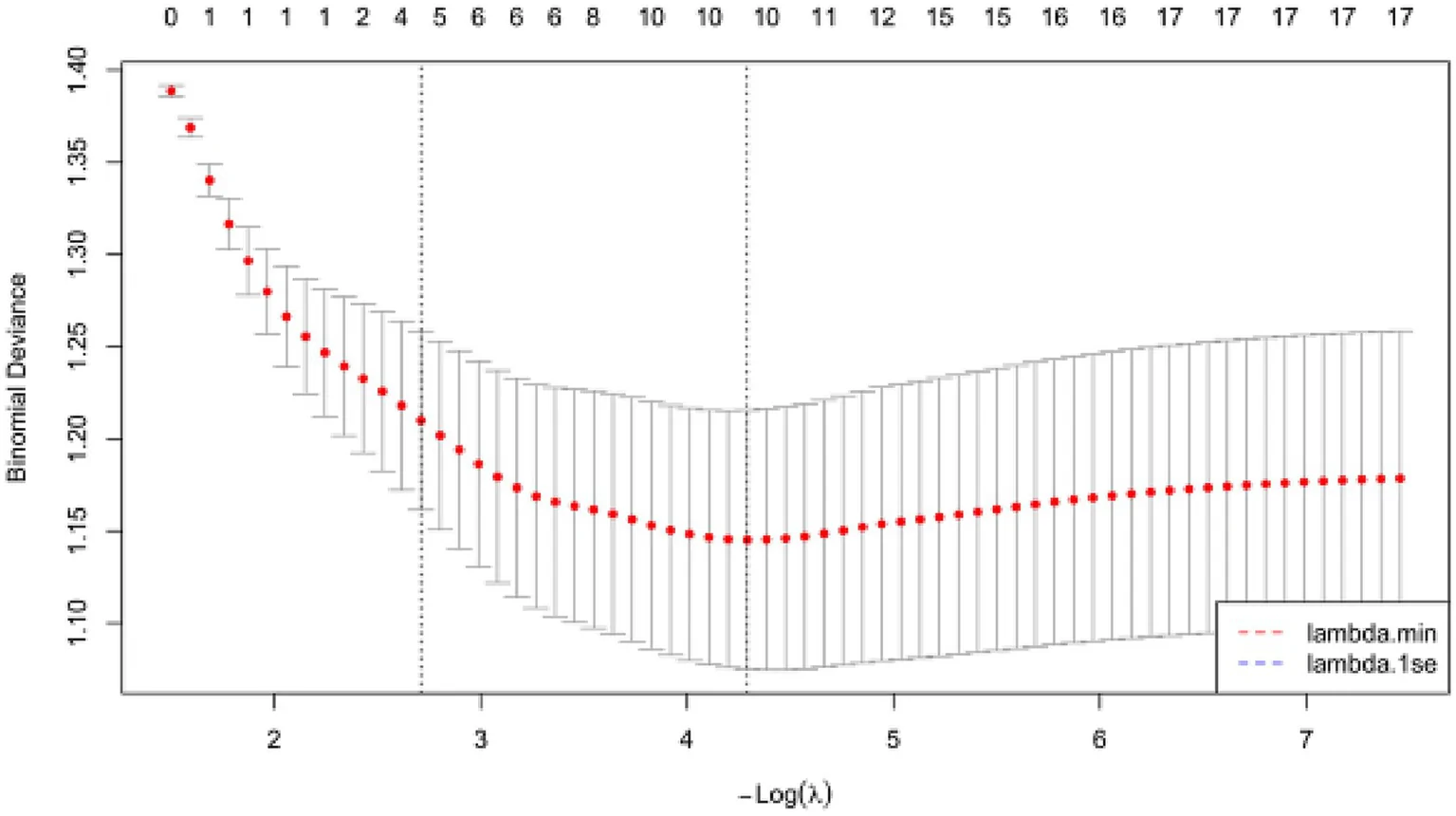
LASSO cross-validation plot.

**Figure 2:**
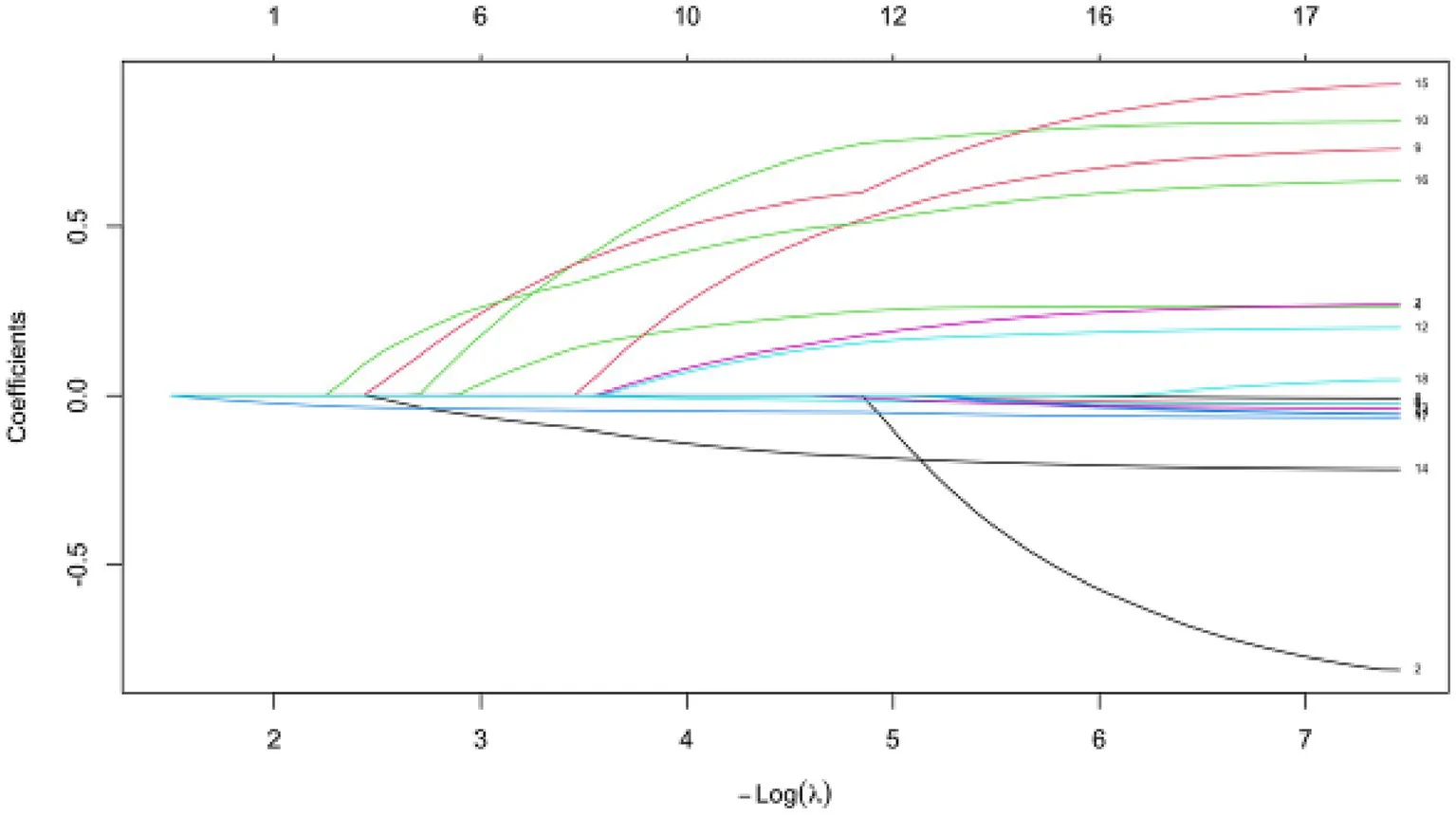
LASSO coefficient path plot.

At the minimum lambda value, LASSO regression retained five variables with non-zero coefficients (Table 3; Figures 1, 2): average monthly household income, eHealth literacy, anxiety, depression, and trust in physicians. This five-variable set constituted the final predictor set and served as the input for all four candidate models.

**Table 3:** Predictor coefficients selected by LASSO regression (λ.min vs. λ.1se).

Variables	Coefficient at lambda.1se	Coefficient at lambda.min
Average monthly household income	0.00436414	0.64927246
eHealth literacy	−0.03584576	−0.16015825
Anxiety	0.11962826	0.54485972
Depression	0.18812753	0.46267089
Trust in physicians	−0.03821380	−0.04801098

### Independent predictors identified by multivariable logistic regression

The five LASSO-selected predictors were entered into a multivariable logistic regression model, reported here as a secondary explanatory analysis to aid interpretation of the prediction model. With 165 events among 336 participants and five candidate predictors, the events-per-variable ratio was 33, which exceeds conventional minimum requirements for logistic model development and reduces the risk of overfitting in the regression model. Trust in physicians, eHealth literacy, anxiety, depression, and average monthly household income were independently associated with decisional regret (all *p* < 0.05; Table 4). Variance inflation factors for all variables were less than 3, indicating no substantial multicollinearity.

**Table 4:** Multivariable logistic regression results (*n* = 336).

Variable	Estimate	Std. error	Wald z	OR (95% CI)	*p* value
Patient death	−0.797	0.609	−1.308	0.451 (0.135, 1.487)	0.191
FCCHL	0.011	0.044	0.258	1.011 (0.928, 1.103)	0.797
Family functioning: good family functioning (Reference)					
Family functioning: moderate-to-severe family dysfunction	0.202	0.466	0.433	1.224 (0.490, 3.086)	0.665
Relationship: sibling (Reference)					
Relationship: spouse	−0.772	0.661	−1.167	0.462 (0.128, 1.768)	0.243
Relationship: child or child-in-law	−0.113	0.635	−0.177	0.893 (0.263, 3.280)	0.859
Relationship: parent	0.545	0.898	0.607	1.725 (0.308, 10.819)	0.544
Relationship: Grandchild	−0.045	1.019	−0.044	0.956 (0.125, 7.216)	0.965
Trust in physicians	−0.064	0.014	−4.451	0.938 (0.911, 0.964)	<0.001
eHealthliteracy	−0.204	0.089	−2.288	0.816 (0.683, 0.971)	0.022*
Household per capita monthly income: ≥10,000 CNY(Reference)					
Household per capita monthly income: <10,000 CNY	0.792	0.348	2.275	2.207 (1.130, 4.446)	0.023*
Anxiety: None (Reference)					
Anxiety: Present	0.914	0.353	2.591	2.495 (1.245, 4.992)	0.010*
Depression: None (Reference)					
Depression: Present	0.679	0.299	2.272	1.973 (1.100, 3.562)	0.023*

*p < 0.05; **p < 0.001.

### Development and validation of the prediction model

In the test set, AUCs across the four candidate models ranged from 0.722 to 0.875. Logistic regression achieved the highest AUC (0.875, 95% CI 0.808–0.942), followed by SVM (AUC 0.814, 95% CI 0.728–0.899), random forest (AUC 0.781), and XGBoost (AUC 0.722). These results indicated that logistic regression provided the strongest discrimination in internal validation.

**Figure 3:**
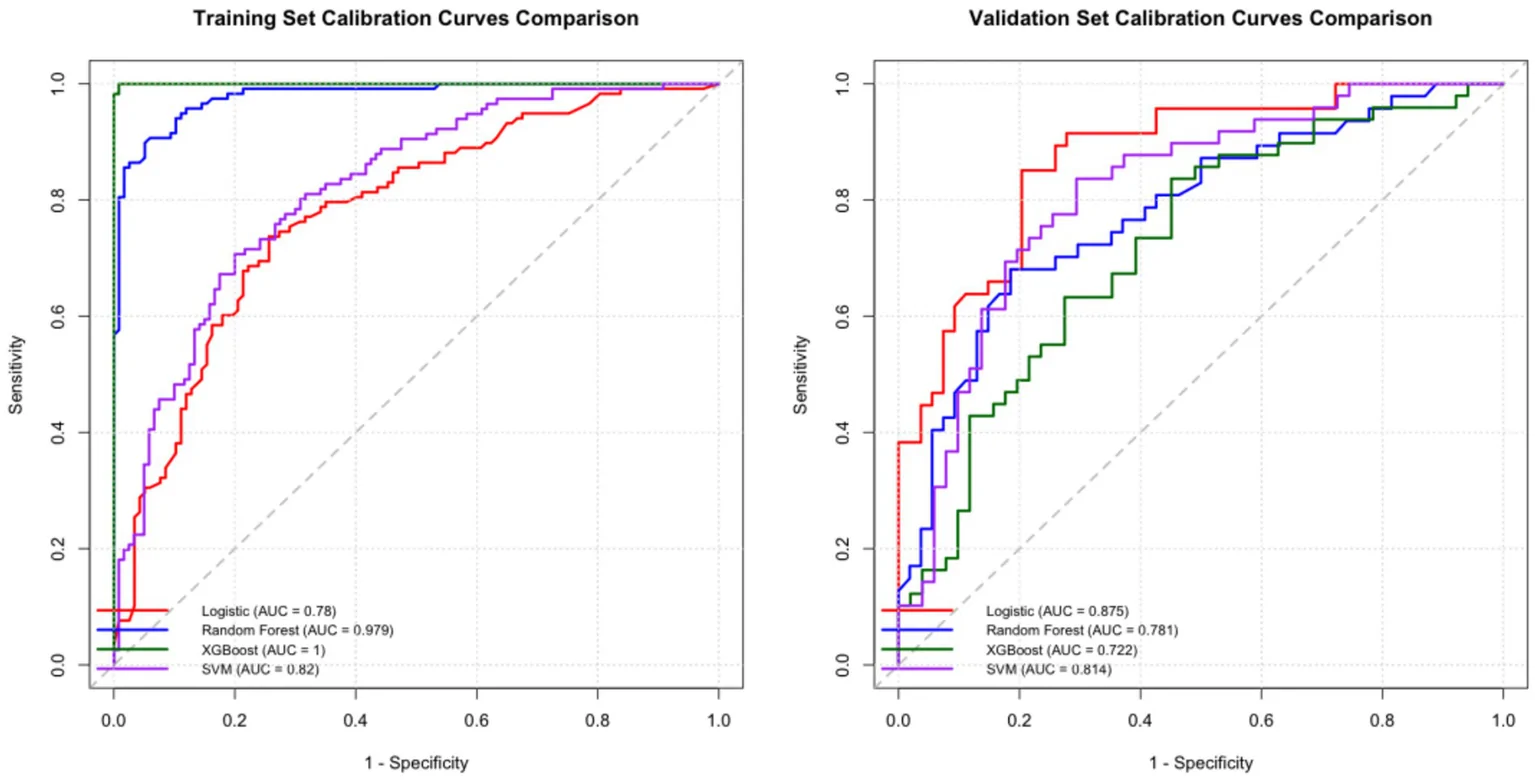
Comparison of ROC curves for four machine-learning models in training and test sets.

When training and test performance were considered together, random forest and XGBoost showed substantially higher AUCs in the training set than in the test set, suggesting possible overfitting. Logistic regression and SVM showed more stable performance across datasets, with logistic regression providing the best overall discrimination (Table 5; Figure 3).

**Table 5:** Predictive performance of four candidate models in the training and test sets.

Model	AUC(95%CI)	Accuracy	Sensitivity	Specificity	F1 score
Training set					
Logistic	0.780 (95% CI: 0.721–0.839)	0.723	0.686	0.761	0.714
RF	0.979 (95% CI: 0.964–0.993)	0.923	0.898	0.949	0.922
XGBoost	0.999 (95% CI: 0.999–1)	0.987	0.983	0.992	0.987
SVM	0.820 (95% CI: 0.767–0.873)	0.750	0.672	0.825	0.726
Test set					
Logistic	0.875 (95% CI: 0.808–0.942)	0.752	0.702	0.796	0.725
RF	0.781 (95% CI: 0.69–0.872)	0.723	0.702	0.741	0.702
XGBoost	0.722 (95% CI: 0.622–0.822)	0.640	0.673	0.608	0.647
SVM	0.814 (95% CI: 0.728–0.899)	0.760	0.735	0.784	0.750

### Calibration performance

**Figure 4:**
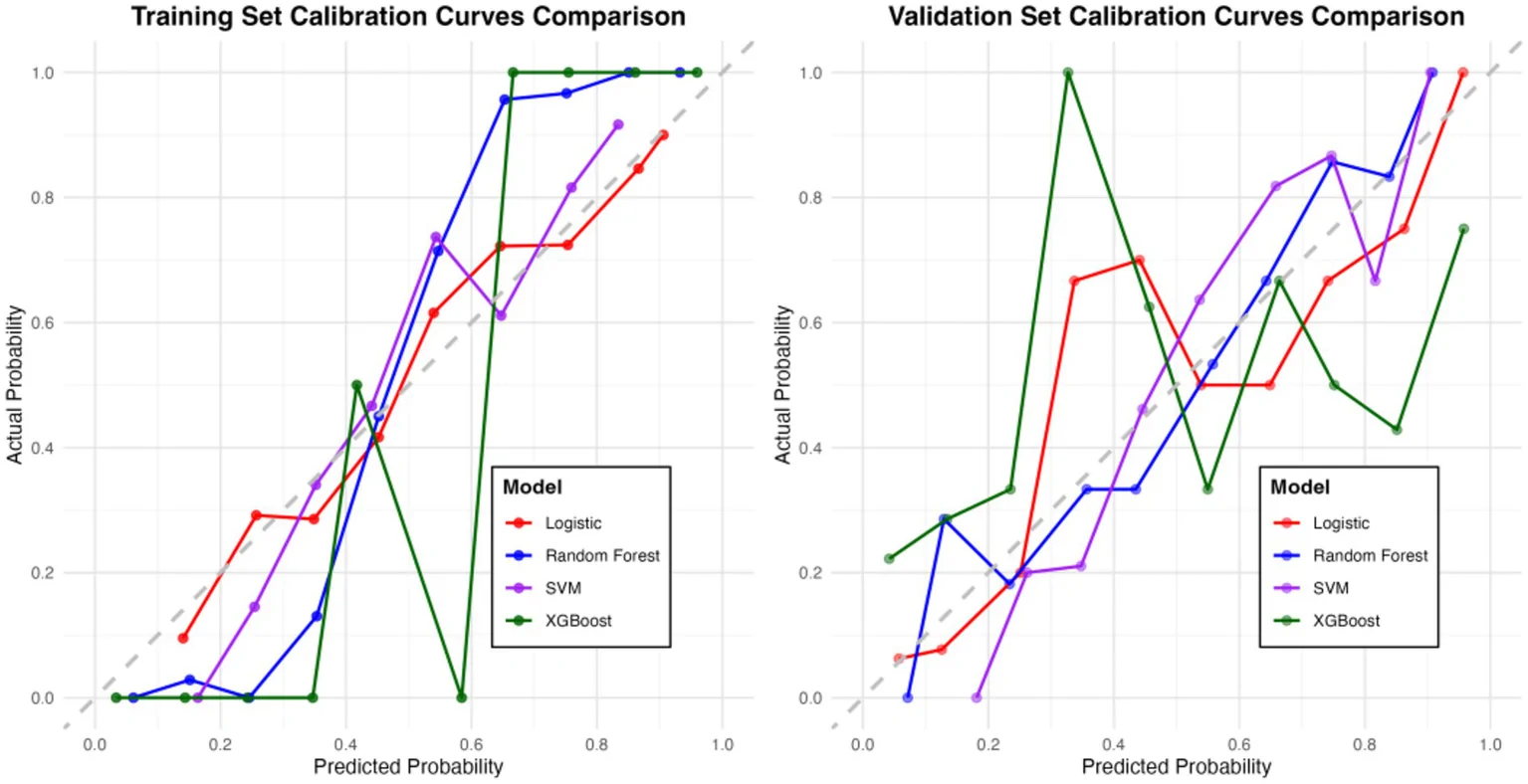
Comparison of calibration plots for four machine-learning models in training and test sets.

Calibration curves (Figure 4) showed differences in agreement between predicted and observed event probabilities. The logistic regression curve was closest to the ideal reference line in both the training and test sets. The SVM model showed the next-best calibration, whereas random forest showed greater deviations in the middle-to-high probability range. XGBoost showed the largest deviation from the reference line. Overall, logistic regression combined good discrimination with the most favorable calibration.

### Clinical utility

**Figure 5:**
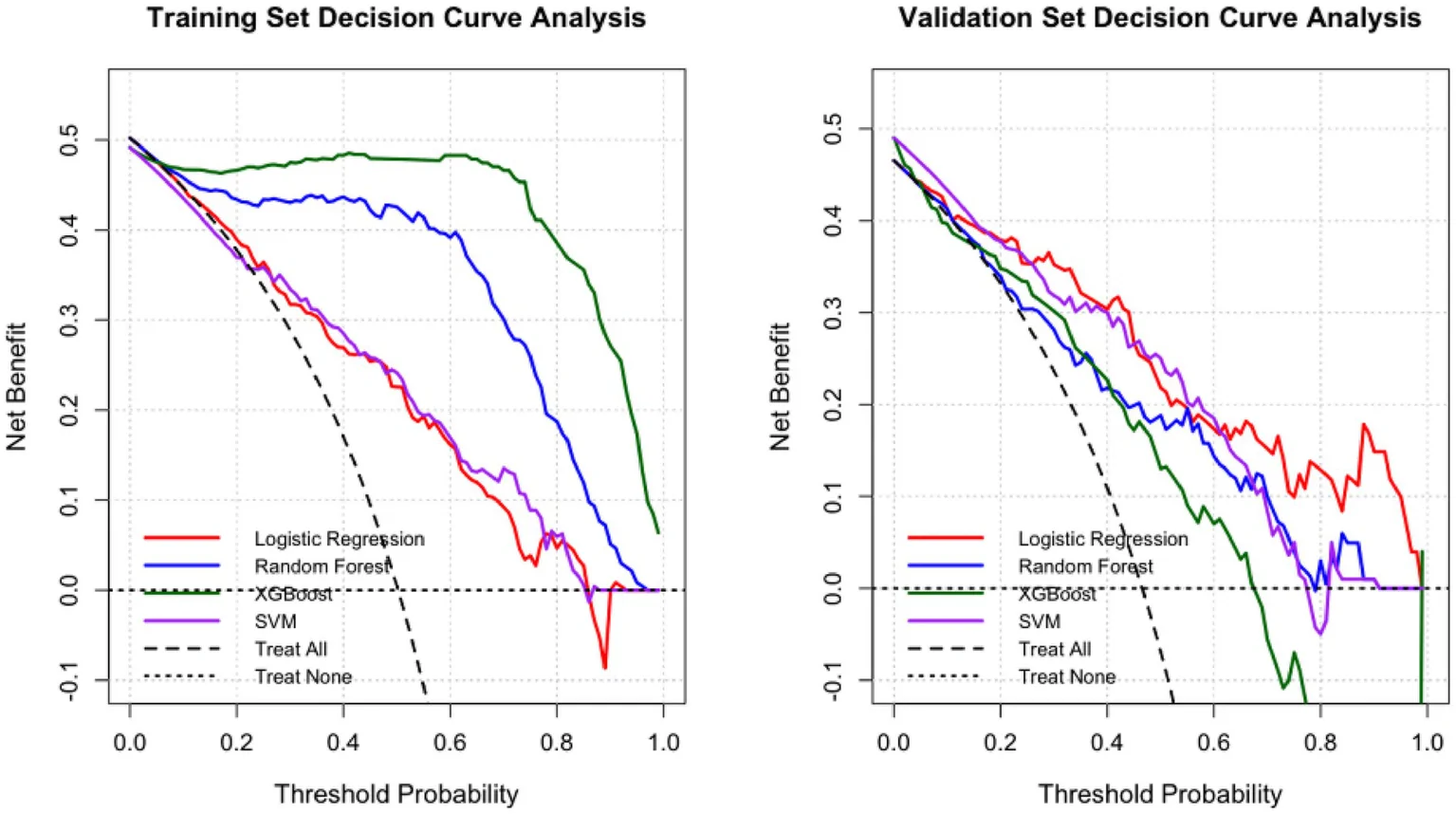
Comparison of decision curves for four machine-learning models in training and test sets.

Decision curve analysis (Figure 5) showed that XGBoost had the highest net benefit across most threshold probabilities in the training set, followed by random forest. In the test set, however, logistic regression maintained a higher and more stable net benefit across a wider range of thresholds. The net benefit of XGBoost declined as the threshold increased and fell below zero at higher thresholds. These results suggested that logistic regression had the most stable clinical utility after internal validation.

### Feature importance analysis

For exploratory model interpretation, SHAP values were calculated for the XGBoost model to estimate each feature’s contribution to the predicted probability of decisional regret. Feature importance was ranked by mean absolute SHAP value, with higher values indicating greater contribution to model output.

**Figure 6:**
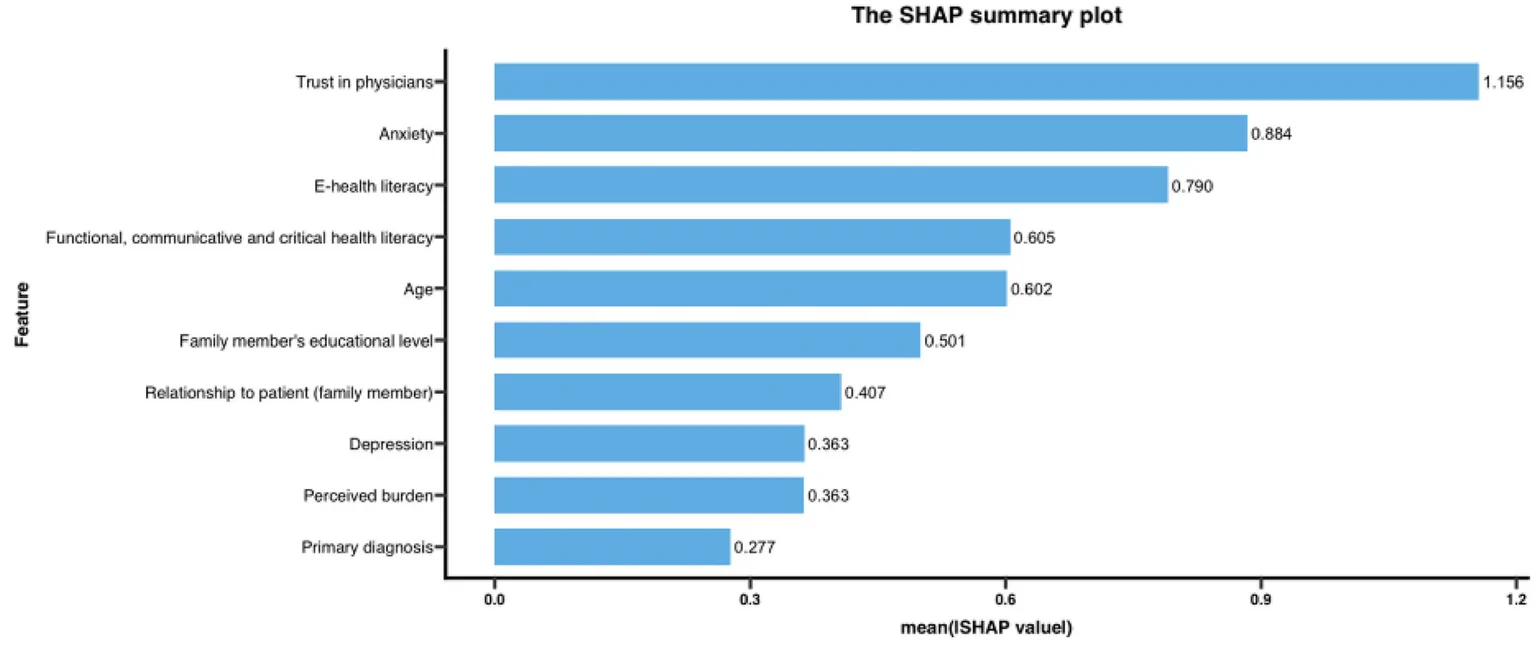
SHAP feature importance ranking based on mean absolute SHAP values.

Figure 6 ranks the predictors by mean absolute SHAP value. Trust in physicians ranked first, followed by anxiety and eHealth literacy. Trust in physicians made the largest contribution.

**Figure 7:**
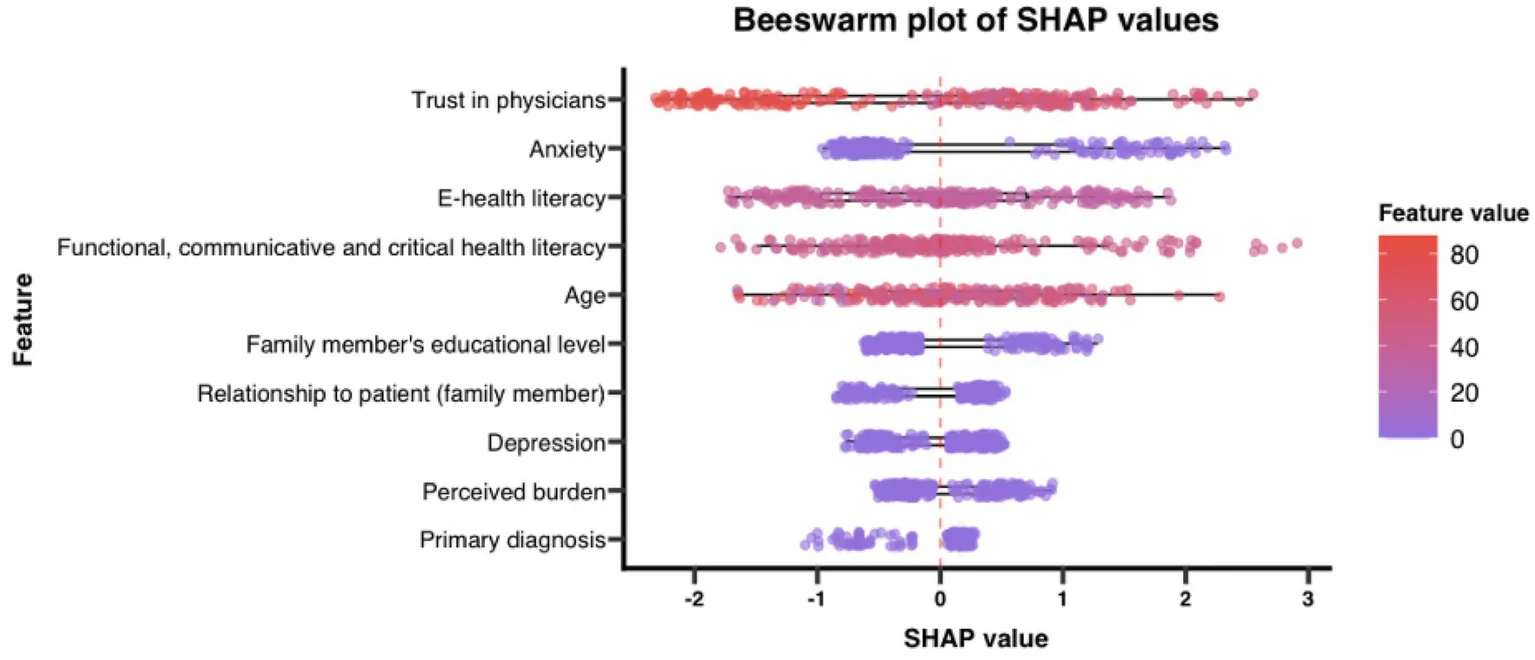
SHAP summary (beeswarm) plot of feature contributions in the XGBoost model.

The SHAP summary plot (Figure 7) similarly indicated that trust in physicians, anxiety, eHealth literacy, FCCHL, and age were prominent contributors. Lower trust in physicians and higher anxiety generally shifted predictions toward a higher probability of decisional regret. Educational level, relationship to the patient, depression, perceived burden, and primary diagnosis also influenced model output to varying degrees.

## Discussion

Guided by a socio-ecological framework, this study examined factors associated with 6-month decisional regret among family surrogate decision-makers of ICU patients and compared four candidate prediction models. Nearly half of participants reported high decisional regret. This finding supports the view that decisional regret is common after ICU surrogate decision-making and requires clinical attention (22, 39). The observed proportion was slightly higher than in some previous reports (40), which may reflect the high uncertainty, time pressure, and emotional burden of ICU decisions. In the Chinese context, family-centered decision-making and expectations of filial responsibility may further intensify self-blame when outcomes are poor or communication is incomplete (41, 42). These findings highlight the need for early identification of high-risk family members and for decision support that addresses both information needs and emotional burden.

Interpretation within the socio-ecological framework. The framework informed which constructs were measured rather than which were retained: candidate variables were chosen to span all four levels, and final predictors were selected empirically by LASSO. The five retained predictors were distributed across three of the four levels — three at the individual level (eHealth literacy, anxiety, depression), one at the healthcare-system level (trust in physicians) and one at the socioeconomic level (average monthly household income). No interpersonal/family-level variable was retained, despite family functioning differing significantly between groups in univariate analysis. One interpretation is that, in the Chinese ICU context, where decision-making authority is concentrated in a single designated surrogate rather than distributed across the family, family functioning may act on regret indirectly through the surrogate’s own affective state, and may therefore contribute little once anxiety and depression are included in the model. This is a hypothesis generated by the present data rather than a tested finding; formal mediation analysis in a larger sample would be required to examine it. Read across levels, the retained predictors suggest that decisional regret is not solely an individual psychological phenomenon: it is shaped jointly by the surrogate’s affective state and information-processing capacity, by the quality of the clinician–family relationship, and by material circumstances, which implies that interventions confined to any single level are unlikely to be sufficient.

Anxiety and depression were important risk factors, suggesting that psychological distress is closely linked to decisional regret. When family members make high-stakes decisions under time pressure and uncertainty, anxiety and depression may impair their ability to receive, understand, and integrate complex medical information (43, 44). After decisions are made, distressed surrogates may be more prone to rumination, self-blame, and counterfactual thinking, which can increase decisional regret (45). This interpretation is consistent with evidence that family members of critically ill patients commonly experience anxiety, depression, and distress (39, 46, 47).

eHealth literacy emerged as an independent protective factor. FCCHL and educational level also contributed in univariate analysis or exploratory SHAP interpretation. Together, these findings suggest that the ability to access, understand, evaluate, and apply health information may shape the quality of surrogate decision-making (48).

Higher information literacy may help surrogates understand illness severity, treatment goals and risks, and support more effective clinician–family communication (49). In contrast, limited information literacy may increase misunderstanding, passive compliance, and later doubts about the decision (50).

Trust in physicians was the most prominent predictor in both multivariable logistic regression and exploratory SHAP analysis. This finding suggests that the physician-family trust relationship may be a central interpersonal factor in decisional regret. In the ICU, physicians provide not only clinical information but also interpretation, guidance, and reassurance during high-stakes decisions (51). Higher trust may help family members accept professional recommendations, reduce uncertainty, and perceive the decision-making process as fair and reasonable (51, 52). Conversely, insufficient trust may increase doubts about communication, treatment necessity, and eventual outcomes, thereby amplifying decisional regret (53).

Lower household income was also associated with increased decisional regret, indicating that socioeconomic resources may shape surrogate decision-making (54). ICU care can impose substantial direct and indirect financial burdens. Families with fewer economic resources may need to weigh potential clinical benefit against financial strain when considering treatment options (54, 55). This constraint may heighten distress during decision-making and lead surrogates to appraise the decision more negatively in retrospect (55).

Several variables, including patient 6-month survival outcome, family functioning, relationship to the patient, and FCCHL, were important in univariate analysis or exploratory SHAP interpretation but were not independent predictors in multivariable logistic regression. Their effects may be indirect, operating through more proximal factors such as emotional state, trust in physicians, and the ability to understand and process information. Alternatively, overlapping explanatory effects may have attenuated their independent contributions after adjustment. These findings suggest that decisional regret should be understood as a multilevel phenomenon rather than as the result of a single isolated factor.

Regarding model performance, random forest and XGBoost achieved high AUCs in the training set, but their discrimination declined in the test set, suggesting possible overfitting. Logistic regression and SVM performed more consistently in internal validation, and logistic regression showed the highest test-set AUC, favorable calibration, and stable clinical net benefit. Several features of this dataset explain why the more complex algorithms did not improve prediction. The training set contained approximately 235 participants, which is modest relative to the sample required for tree ensembles to estimate interactions reliably; in this regime the bias–variance trade-off favors simpler models. The five retained predictors were continuous scale scores whose relationships with decisional regret appear monotonic, so a linear-additive specification was close to correct and the flexible algorithms had little additional structure to exploit, adding variance without reducing bias. The predictor set was also small, whereas the advantages of machine learning typically emerge in higher-dimensional data. Consistent with this interpretation, XGBoost achieved a training AUC of 0.999 but only 0.722 in the test set, the characteristic signature of variance rather than of superior signal extraction. We therefore interpret these findings as specific to this dataset—flexible algorithms offered no advantage where the predictor–outcome relationships were few in number and approximately linear—rather than as evidence that simpler models are generally preferable.

For clinical use, a prediction model must provide not only discrimination but also calibrated risk estimates and practical value. In this study, logistic regression offered the most balanced performance and may be the most suitable approach for risk screening.

Exploratory SHAP analysis added interpretability to the XGBoost model and helped visualize how individual predictors contributed to model output. The analysis emphasized trust in physicians, anxiety, and eHealth literacy as key contributors. These results were broadly consistent with multivariable regression and suggest that decisional regret is more closely related to modifiable psychological and communication-related factors than to demographic characteristics alone. This finding supports future interventions that strengthen clinician-family communication, provide tailored information, and address psychological distress.

### Strengths and limitations

This study has several limitations. First, the model was developed and internally validated in a single cohort recruited from one tertiary hospital, and no external validation was performed. Four consequences follow. (i) Calibration is the most vulnerable property. High decisional regret occurred in 49.11% of this cohort, a higher proportion than in some previous reports; because the model intercept is calibrated to this baseline risk, predicted probabilities would be systematically too high in settings where regret is less frequent, even if the ranking of individuals by risk were preserved. (ii) Case-mix and spectrum may differ. Participants were recruited from five ICUs of a 4,300-bed university-affiliated hospital; the distribution of predictors such as household income and eHealth literacy, and their relationship with regret, may differ in secondary or rural hospitals. (iii) The decision-making context is specific. In China, uptake of advance directives is low and treatment decisions are commonly led by family surrogates under strong norms of filial responsibility; where advance directives are common or the surrogate role is more formalized, the decisional burden differs and predictors such as trust in physicians may carry different weight. (iv) Measurement equivalence is not guaranteed: the model assumes the same instrument versions and the same DRS threshold of 25, which may not perform equivalently across languages and cultures. In terms of the TRIPOD framework, this study reports model development with internal validation only. The model is therefore appropriate for research and hypothesis generation, and should not be used to guide individual clinical decisions until it has been externally validated and, where necessary, recalibrated. Second, psychological status was assessed only once, within 3 days of ICU discharge, whereas the outcome was measured 6 months later. Anxiety and depression may change substantially over this interval, and a single baseline measurement is therefore a noisy proxy for psychological state at the time of outcome assessment; such non-differential misclassification would be expected to attenuate the observed associations, making our estimates conservative rather than inflated. Third, events occurring during follow-up, in particular patient death, may affect both psychological status and decisional regret. Fourth, decisional regret was assessed retrospectively by telephone at 6 months, so mood-congruent recall may have influenced how the earlier decision was appraised. Repeated measurement of psychological status during follow-up would allow trajectories rather than a single baseline value to be modeled, and we recommend this design for future studies. Third, this observational study identified predictors rather than causal determinants, and variable-importance rankings should not be interpreted as causal effect sizes. Fourth, the prediction models were internally validated using a train-test split only. This model has only completed internal validation and should not be applied to actual clinical decision making until external validation is completed. At the same time, the study scenario is a cultural background of single-center and family-led decision-making in China, and the model needs to be recalibated when it is promoted to regions with high prevalence of advance directives. Future studies should use longitudinal designs, external validation, and intervention studies to examine whether risk-based decision support can reduce decisional regret among ICU family surrogate decision-makers. And external validation across hospitals of differing tier and region is the necessary next step.

## Conclusion

Decisional regret was common among ICU family surrogate decision-makers. Trust in physicians, eHealth literacy, household income, anxiety, and depression were key predictors. Among the candidate models, logistic regression provided the best balance of discrimination, calibration, and clinical utility in internal validation. These findings suggest that ICU teams should identify family surrogate decision-makers who may need additional support, especially those with limited eHealth literacy, financial disadvantage, anxiety, depression, or low trust in physicians. Stratified support may include clearer information delivery, written or visual decision aids, consistent communication across clinicians, and timely psychological referral. Further external validation is needed before the model is implemented in routine practice.

## Data Availability

The raw data supporting the conclusions of this article will be made available by the authors, without undue reservation.
