# Supplementary material for: Predicting 6-month decisional regret among family surrogate decision-makers of ICU patients: development and internal validation of prediction models
Source: Front Public Health. 2026 Sep 3;14:1928359. doi: 10.3389/fpubh.2026.1928359 (PMC13581493; doi:10.3389/fpubh.2026.1928359)
Supplement: Supplementary file 1 [file Supplementary_file_1.docx]

**STROBE Reporting Checklist Mapping**

Author-completed location map for the observational component

This document maps the manuscript to STROBE reporting domains. It uses brief author-facing item labels rather than reproducing the full official checklist. Use the official EQUATOR/STROBE file if the submission system requires the journal-specific checklist template.

| **STROBE domain** | **What is reported** | **Location in manuscript** |
| --- | --- | --- |
| Title/Abstract | Study design, setting, sample, variables, outcomes, model methods, and key results | Title; Abstract |
| Background/Rationale | Scientific background and rationale for studying decisional regret in ICU surrogate decision-makers | Introduction, paragraphs 1-4 |
| Objectives | Study objectives and prediction-model aim | End of Introduction; Abstract Aim/Objectives |
| Study Design | Single-center observational study; five ICUs; study period | Methods: Study design and setting |
| Setting | Tertiary university-affiliated hospital in Hangzhou, China; October 2023-October 2024 | Methods: Study design and setting |
| Participants | Eligibility, inclusion/exclusion criteria, consent, withdrawal | Methods: Study Population |
| Variables | Outcome, predictors, sociodemographic and psychosocial measures | Methods: Measurements; Data collection |
| Data Sources/Measurement | Validated scales and data collection timing | Methods: Measurements; Data collection |
| Bias | Pilot survey, trained investigators, standardized instruments; residual recall/selection bias noted | Methods: Data collection; Discussion: Strength and limitation |
| Study Size | 400 questionnaires collected; 336 included after exclusions | Results: Baseline characteristics |
| Quantitative Variables | DRS cutoff, scale scores, categorical variables, model predictors | Methods: Measurements; Statistical analysis |
| Statistical Methods | Univariate tests, LASSO, LR/RF/SVM/XGBoost, calibration, DCA, SHAP | Methods: Statistical analysis; Model development and validation |
| Participants Flow | Questionnaires collected and excluded; final analytic sample | Results: Baseline characteristics |
| Descriptive Data | Baseline characteristics by high/low regret group | Results: Table 1 |
| Outcome Data | High vs low decisional regret at 6 months | Results: Baseline characteristics |
| Main Results | Predictors and model performance | Results: Univariate analysis; Model performance; Table 2; Figures 3-7 |
| Other Analyses | Calibration, decision curve analysis, SHAP interpretation | Results: Calibration performance; Clinical utility; Feature importance analysis |
| Key Results | Trust, eHealth literacy, income, anxiety, depression; LR best overall performance | Discussion opening; Conclusion |
| Limitations | Single center, sample size, recall bias, prediction not causality, no external validation | Discussion: Strength and limitation |
| Interpretation | Clinical interpretation and nursing relevance | Discussion; Conclusion |
| Generalisability | Need for larger multicenter/external validation | Discussion: Strength and limitation |
| Funding | Funding source declared | Title page; Funding Declaration |
